# Selenium and the 15kDa Selenoprotein Impact Colorectal Tumorigenesis by Modulating Intestinal Barrier Integrity

**DOI:** 10.3390/ijms221910651

**Published:** 2021-09-30

**Authors:** Jessica A. Canter, Sarah E. Ernst, Kristin M. Peters, Bradley A. Carlson, Noelle R. J. Thielman, Lara Grysczyk, Precious Udofe, Yunkai Yu, Liang Cao, Cindy D. Davis, Vadim N. Gladyshev, Dolph L. Hatfield, Petra A. Tsuji

**Affiliations:** 1 Department of Biological Sciences, Towson University, Towson, MD 21252, USA; Jessica.Canter@usda.gov (J.A.C.); sgalinn1@jhmi.edu (S.E.E.); kristin.peters718@gmail.com (K.M.P.); nthielman@lecom.edu (N.R.J.T.); lara@grysczyk.de (L.G.); pudofe1@students.towson.edu (P.U.); 2 Mouse Cancer Genetics Program, Center for Cancer Research, National Cancer Institute, National Institutes of Health, Bethesda, MD 20892, USA; carlsonb@dc37a.nci.nih.gov (B.A.C.); hatfielddolph@gmail.com (D.L.H.); 3 Lake Erie College of Osteopathic Medicine, Erie, PA 16509, USA; 4 Genetics Branch, Center for Cancer Research, National Cancer Institute, National Institutes of Health, Bethesda, MD 20892, USA; yuyun@mail.nih.gov (Y.Y.); caoli@mail.nih.gov (L.C.); 5 Office of Dietary Supplements, National Institutes of Health, Bethesda, MD 20817, USA; Cindy.Davis2@usda.gov; 6 Brigham and Women’s Hospital, Harvard Medical School, Boston, MA 02215, USA; vgladyshev@rics.bwh.harvard.edu

**Keywords:** Selenof, selenium, selenoprotein, colon cancer, inflammation, barrier integrity

## Abstract

Selenoproteins play important roles in many cellular functions and biochemical pathways in mammals. Our previous study showed that the deficiency of the 15 kDa selenoprotein (*Selenof*) significantly reduced the formation of aberrant crypt foci (ACF) in a mouse model of azoxymethane (AOM)-induced colon carcinogenesis. The objective of this study was to examine the effects of *Selenof* on inflammatory tumorigenesis, and whether dietary selenium modified these effects. For 20 weeks post-weaning, Selenof-knockout (KO) mice and littermate controls were fed diets that were either deficient, adequate or high in sodium selenite. Colon tumors were induced with AOM and dextran sulfate sodium. Surprisingly, KO mice had drastically fewer ACF but developed a similar number of tumors as their littermate controls. Expression of genes important in inflammatory colorectal cancer and those relevant to epithelial barrier function was assessed, in addition to structural differences via tissue histology. Our findings point to *Selenof*’s potential role in intestinal barrier integrity and structural changes in glandular and mucin-producing goblet cells in the mucosa and submucosa, which may determine the type of tumor developing.

## 1. Introduction

Colon cancer remains the second leading cause of cancer-related deaths in the United States with an estimated 104,270 new cases and 52,980 deaths in 2021 [1]. One of the earliest indicators of colorectal cancer development is the formation of aberrant crypt foci (ACF), which are pre-neoplastic lesions in the form of abnormal tube-like glands in the colorectal lining tissue. The number of ACF is thought to have a strong relationship with the number of tumors formed in the colon [2], with about 20–30% of ACF predicted to develop into tumors. Intestinal inflammation is known to promote colorectal cancer through a variety of different mechanisms [3,4,5]. These include pro- and anti-inflammatory cytokines, oxidative stress, and even the composition of the intestinal microbiota [6]. Many of these mechanisms are thought to be modulated by dietary selenium [7,8,9].

Selenium is an essential trace mineral found in many foods commonly consumed in the U.S. diet as organic forms, such as selenocysteine and selenomethionine, and inorganic forms, such as sodium selenite [10,11]. Much of selenium’s role in health and disease has been attributed to its incorporation into selenoproteins, which are encoded by 25 different genes in humans and 24 genes in mice [12]. Selenoproteins play crucial roles in cellular processes such as DNA synthesis, apoptosis, and protection from oxidative damage [13,14,15]. Previous studies have shown an inverse relationship between dietary selenium levels and the risk of colon cancer, as well as the functional role of selenoproteins in colorectal cancer (reviewed in [16]).

Among the many selenoproteins, the 15 kDa selenoprotein (*Selenof*, formerly known as *Sep15* or *Sel15*) is expressed in high levels in liver, prostate, kidney, testis, and brain. It is furthermore expressed at very high levels in colon cancer cells [17,18]. SELENOF’s molecular function appears to be in quality control of oxidative protein folding in the endoplasmic reticulum and signaling in the cellular misfolded protein response [15,19,20,21,22]. Recently, a function as a molecular gate keeper and redox quality control role for immunoglobulins has been described [23]. However, the physiological functions of SELENOF and its role in human health, particularly in inflammation and colorectal cancer, are not well understood. Human and mouse colon cancer cell lines with a targeted downregulation of the *Selenof* gene have been generated previously. Our findings suggested a role for *Selenof* in cell replication, invasion and metastasis, as well as a potential regulation of interferon (IFN)-γ-mediated signaling pathways [17,18,24]. To investigate the role of *Selenof* in health and disease in vivo, a Selenof-knockout (Selenof-KO) model was created using C57BL/6 mice. Systemic SELENOF expression was inhibited in these mice by the targeted insertion of a transcriptional terminator in exon 2 of the *Selenof* gene [20,25]. To create littermate controls for comparison with these KO mice, heterozygous mice were backcrossed to create a pseudo-wild type (WT) mouse group, as well as a Selenof-KO mouse group from the same set of parents. This preserved any genetic background as well as environmental factors that may influence the development of the animals. These Selenof-KO mice have a typical C57BL/6 morphology with no visible phenotypic abnormalities. They do, however, appear to have increased levels of inflammation in the form of elevated serum interferon (IFN)-γ expression [26], and develop cataracts early in life [20]. Despite the apparent increase in basal inflammation, we showed in a previous study that these Selenof-KO mice produce significantly fewer ACF than littermate control mice when exposed to the colon-specific chemical carcinogen azoxymethane (AOM) [26]. These results agreed with the findings in cell culture, where a targeted down-regulation of *Selenof* expression resulted in a reversal of the colon cancer phenotype: reduced cell proliferation, reduced ability to grow anchorage-independently, with a concomitant increase in expression of IFN-γ-regulated guanylate binding protein (GBP)-1 [17,18,26]. In vivo, the effects were modified by dietary selenium, where Selenof-KO mice showed a modest increase in the number of ACF under conditions of selenium-deficiency [26].

In this subsequent study, we were interested to assess whether Selenof-KO mice were also protected against the development of tumors in an inflammatory colon tumorigenesis model, the possible impact dietary selenium had, and whether the colon cancer-specific signaling mechanisms impacted by *Selenof* could be further elucidated. Therefore, Selenof-KO mice and their wildtype (WT) littermates were injected with AOM and exposed to the inflammatory agent, dextran sulfate salt (DSS), and were compared to untreated controls. The addition of DSS allowed us to observe tumors formed, in addition to the ACF expected from AOM-treatment alone. The number of ACF, tumor incidence and mass, gene expression of cell signaling pathways, and production of serum cytokines were analyzed to examine responses in mice from each group. Various factors thought to contribute to the development of inflammatory colon cancer, including the enzymes responsible for bioactivation of the carcinogen, inflammatory cytokines, and measures of the barrier integrity of the intestinal epithelium, were investigated. The results of this study contribute to understanding the role of *Selenof* in the development of inflammatory colon cancer. This knowledge may be useful in further investigation into human health, where functional single nucleotide polymorphisms for *SELENOF* have been reported [27,28,29]. The allele frequency of such single nucleotide polymorphisms in the *SELENOF* gene appear to differ by ethnicity [27]. Because the identity of nucleotides at the polymorphic sites has been shown to influence selenocysteine insertion during translation in a selenium-dependent manner, differentially expressed *SELENOF* may influence health outcomes or susceptibility to cancer in specific populations.

## 2. Results

Post-weaning, male Selenof-KO and WT littermate mice were maintained on a Torula yeast-based diet (Teklad Harlan Laboratories, Madison, WI, USA) with deficient (measured amounts were 0.02 µg/g diet), adequate (0.1 µg/g diet) or high (2.0 µg/g diet) levels of sodium selenite for the duration of the study. Animals were injected subcutaneously with either AOM (10 mg/kg) or saline at six weeks of age, and subsequently subjected to two one-week rounds of drinking water with or without 2% DSS, respectively (Figure S1). Mice were sacrificed after 20 weeks, and tissue samples and serum were collected.

### 2.1. Growth Metrics

All mice were weighed upon entry into the study, twice weekly thereafter, and sacrificed after 20 weeks. Weight gain (Figure S2a,b) was calculated by subtracting the mass determined at entry into the study from the final mass determined at sacrifice, and analyzed with a 2-way ANOVA followed by Tukey’s multiple comparisons (N = 10–12/group). Under selenium-deficient conditions, control Selenof-KO mice gained significantly more weight (mean weight gain = 29.17 g) than control WT mice (mean weight gain = 14.76 g; *p* < 0.0001), and also compared to control Selenof-KO mice on selenium-adequate (*p* = 0.0009) or high selenium (*p* = 0.0012) diets. AOM/DSS treatment affected all mice, as generally a lower weight gain was observed (Figure S2b). Surprisingly, only dietary selenium (ANOVA, *p* < 0.0001) but not *Selenof* genotype (ANOVA, *p* = 0.1094) affected weight gain under these conditions, with a higher weight gain observed in WT mice on a high selenium diet compared to WT mice on a selenium-deficient diet (Tukey’s, *p* < 0.001).

Absolute colon length from anus to caecum was greatest in Selenof-KO control mice, which correlated with a greater body mass of these animals. Colon length (cm) was normalized against body mass (g), which was determined at sacrifice to compare relative colon length of the animals. Analyses of these data did not indicate any statistically significant differences in weight-normalized colon lengths among control animals (Figure S2c). However, dietary selenium affected AOM/DSS-treated animals (ANOVA, *p* = 0.0003), wherein WT mice on a selenium-deficient diet had a slightly (*p* = 0.0329) greater relative colon length in comparison to WT mice on a high selenium diet. No such increase was observed in Selenof-KO mice. Spleen mass (g) was also determined and expressed relative to total body mass (g). A trend of higher relative spleen mass was observed in Selenof-KO animals exposed to AOM/DSS (ANOVA, *p* = 0.0208 for genotype; Figure S2f), though post hoc analyses failed to reach statistical significance for individual comparisons. Overall, Selenof-KO mice and their WT littermate controls were very similar in terms of growth metrics, whereas dietary selenium levels appeared to exert a modest influence.

### 2.2. Aberrant Crypt Foci Formation and Tumorigenesis

Although only a small percentage of ACF are thought to become malignant [30], ACF are much more prevalent in colorectal cancer cases and therefore often regarded as biomarkers for colon tumors [31]. None of the untreated (control) Selenof-KO mice spontaneously developed tumors; however, one Selenof-KO mice on the selenium-deficient diet spontaneously developed one ACF, though no ACF were detected among Selenof-KO mice on selenium-adequate or high-selenium diets (Table 1). Among the untreated (control) WT mice, no spontaneous ACF were detected in the eight mice on selenium-deficient diets (Table 1). However, 25% of mice on selenium-adequate diets developed three and four ACF, respectively, and 22% of mice on high-selenium diets developed one ACF each. Furthermore, one WT mouse on selenium-adequate diet spontaneously developed two small tumors.

**Figure 1:**
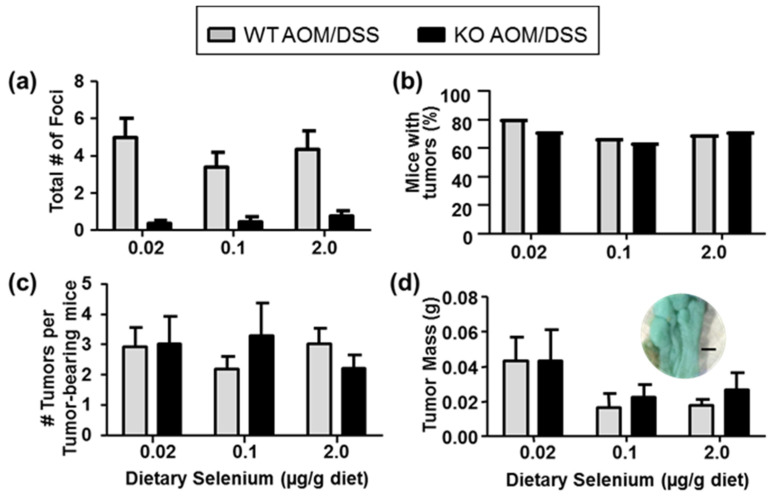
Aberrant crypt foci (ACF) and tumor formation modulated by *Selenof* genotype and dietary selenium. (**a**) AOM/DSS-treated Selenof-KO mice formed fewer chemically induced ACF than WT littermates (two-way ANOVA, genotype *p* < 0.0001), independent of dietary selenium levels. (**b**) Development of macroscopic tumors, and (**c**) number of tumors per colon in Selenof-KO and WT mice did not differ. (**d**) Tumor mass appeared slightly modified by dietary selenium (two-way ANOVA, *p* = 0.117); insert shows stained colon tissue of WT mouse with tumors on selenium-deficient diet (size bar indicates 1 mm).

Among the AOM/DSS-treated mice, as expected based on our previous observations in AOM-treated mice [26], Selenof-KO mice also formed significantly fewer pre-neoplastic lesions than WT mice when exposed to AOM/DSS, regardless of dietary selenium levels (Table 1, Figure 1a). ACF were detected in 86.7% of WT mice on selenium-deficient diet, 72.2% on selenium-adequate diet, and 76.9% on high-selenium diet, respectively. In contrast, only 28.6%, 27.3%, and 42.9% of Selenof-KO mice developed ACF, respectively. As anticipated, we found that 70–80% of the WT mice exposed to AOM/DSS developed colorectal tumors, with a slightly greater number of mice developing tumors under selenium-deficient conditions. Surprisingly, a similar number of Selenof-KO mice developed tumors, regardless of dietary selenium levels (Figure 1b). Similarly, a comparison of the number of tumors per tumor-bearing animal showed no statistically significant differences between Selenof-KO and WT mice, regardless of dietary selenium levels (Figure 1c). Furthermore, no differences were detected in absolute tumor mass among the various groups. However, it is interesting to note that the average tumor mass in all animals on a selenium-deficient diet was 50–100% greater than in animals with adequate or high selenium levels (*p* > 0.05; Figure 1d), providing support that adequate selenium consumption may be helpful in mitigating diseases such as colorectal cancer (reviewed in [16]). Therefore, it appears that while lack of *Selenof* still resulted in significantly decreased numbers of pre-neoplastic lesions, the number or size of actual tumors formed was not influenced by *Selenof* expression.

### 2.3. Expression and Catalytic Activity of Carcinogen-Activating Enzymes

The formation of chemically induced ACF is well established. Bioactivation of AOM occurs primarily in the liver through hydroxylation via hepatic cytochrome P450 (CYP) 2E1. Subsequently, methylazoxymethanol is formed, which can lead to DNA guanine alkylation and formation of persistent DNA adducts in the colon. Alcohol dehydrogenase (ADH1) and UDP-glucuronosyltransferases (UGT) may additionally modify the activation pathway in liver and colon tissues [32,33]. As a result of AOM metabolism, early neoplastic lesions, ACF, appear in colons.

**Figure 2:**
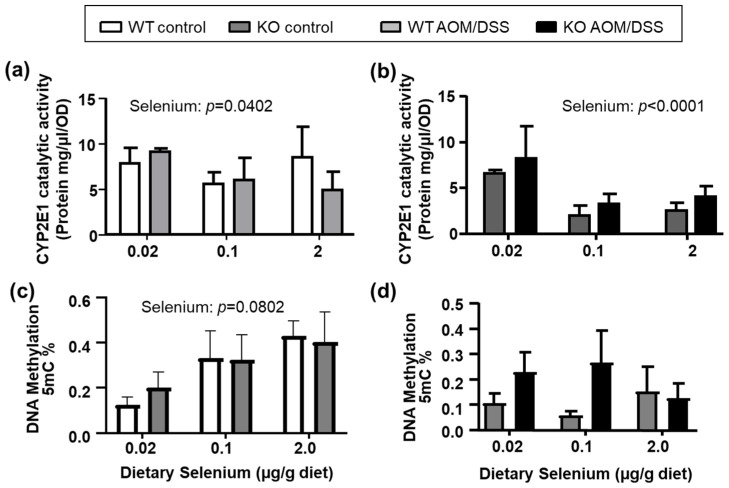
Hepatic AOM-metabolism. Catalytic activity of CYP2E1 in hepatic microsomes was affected by dietary selenium levels, but not by the *Selenof* genotype in (**a**) untreated or (**b**) AOM/DSS treated animals. (**c**) Global 5-mC DNA methylation in liver increased with dietary selenium in control animals. (**d**) AOM/DSS-treated animals displayed greater variability, but no statistically significant differences. Mean (N = 4–8) + SEM, analyzed by 2-way ANOVA, followed by Tukey’s post hoc analyses.

The catalytic activity of CYP2E1 in liver microsomes (two-way ANOVA, N = 8/group) suggested that dietary selenium affected catalytic activity of CYP2E1 in both untreated (Figure 2a) and AOM/DSS-treated (Figure 2b) animals, with a visible decrease in CYP2E1 activity at adequate and high selenium levels. As part of the bioactivation of AOM via CYP2E1 and ADH1, the oxidized product, methylazoxyformaldehyde, through further modifications yields the methyldiazonium ion. In turn, this ion is thought to methylate DNA bases in AOM- and methylazoxymethanol-target tissues and elicit oxidative stress [33,34]. Dietary selenium has also been shown to affect DNA-methylation in various in vitro [35] and in vivo models [35,36,37], and the effects of *Selenof* status on DNA methylation was unknown. Therefore, we also assessed the global DNA methylation (Figure 2c,d) in hepatic tissues of Selenof-KO mice and WT littermates. As expected based on other studies [37,38], global DNA methylation in liver tissues positively correlated with increasing dietary selenium in our animals albeit differences not being statistically significant (Figure 2c). This trend was no longer detectable in AOM/DSS-treated animals (Figure 2d). Additionally, statistically significant differences between Selenof-KO and WT mice were not detected. We also assessed mRNA expression of hepatic *Cyp2e1*, *Adh1,* and DNA methyltransferase 1 (*Dnmt1*) and 3a (*Dnmt3a*) (Figure S3). Although *Adh1* expression appeared to increase with dietary selenium (2-way ANOVA, *p* = 0.0041, Figure S3), mRNA expression of AOM-metabolizing enzymes remained largely unaffected by genotype and dietary selenium in control or AOM/DSS-treated animals. Therefore, it appears that overall, the ability to metabolize AOM via the hepatic CYP2E1 pathway only minimally differs between mice with and without functional SELENOF, with an interesting effect of dietary selenium observed.

Because the metabolism of AOM may continue in colon tissues, where CYP2E1, ADH1 and UGT isoforms process metabolites generated in the liver [32], expression of these genes was assessed in colon scrapes of control animals and in tumors of AOM/DSS-treated animals (Figure S4). The mRNA expression of *Cyp2e1* in colon scrapes of WT and Selenof-KO mice was over 1000-fold less than observed in liver, and were at the limit of detection for AOM/DSS-treated mice on selenium-deficient diets, so we were unable to assess catalytic activity of CYP2E1 in colon tissues. *Cyp2e1* mRNA expression was modestly decreased at high dietary selenium levels in untreated control animals (Figure S4a), and appeared to positively correlate with increasing dietary selenium in colon tumors of AOM/DSS-treated animals (Figure S4b). However, no statistically significant differences were detected for mRNA expression of *Cyp2e1*, *Adh1* (Figure S4c,d) or *Ugt1a* (Figure S4e) in colons among mice with and without *Selenof* expression. *Ugt1a* mRNA levels were below levels of detection in tumors of AOM/DSS-treated mice. Therefore, it appears that the general ability to metabolize AOM does not differ between WT and Selenof-KO mice.

### 2.4. Serum Inflammatory Markers

Our previous study suggested an increased basal inflammatory state in mice lacking *Selenof* expression [26], especially as it relates to interferon (IFN)-γ and interleukin (IL)-6. Therefore, serum levels of several inflammatory markers were determined using an ELISA-based immunoassay (Figure S5). Overall, modest increases in circulating IFN-γ, IL-10, IL-12p70, IL-1β, C-X-C motif ligand 1 (CXCL1), and tumor necrosis factor (TNF)-α were observed in WT and Selenof-KO mice when treated with AOM/DSS in comparison to their untreated controls, respectively. This suggests, that AOM/DSS treatment resulted in a general increase in production of inflammatory cytokines as would be expected. Systemic *Selenof* expression also appeared to impact production of some circulating serum cytokines. Levels of IL-10 (Figure S5c, *p* < 0.05) and IL-1β (Figure S5g, *p* > 0.05) decreased in control Selenof-KO mice, but only under selenium-deficient conditions. Levels of IL-12p70 (Figure S5f, *p* < 0.05) significantly decreased in AOM/DSS treated mice, but only under selenium-deficient conditions, making interpretations difficult. Therefore, it appears that, as expected, both dietary selenium and AOM/DSS treatment impact serum levels of cytokines relevant to inflammation and cancer. However, mice without *Selenof* expression may be showing some sensitivity to selenium-deficiency, where IL-10 was detected in lower amounts in Selenof-KO control mice compared to their WT littermates. Given that IL-10 plays a dual role in tumor development, these results remain inconclusive. Thus, we continued to focus on tissue-specific differences between WT and Selenof-KO mice that might explain the differences in ACF and tumor burden.

### 2.5. Colorectal Cancer Cell Signaling Pathways

**Figure 3:**
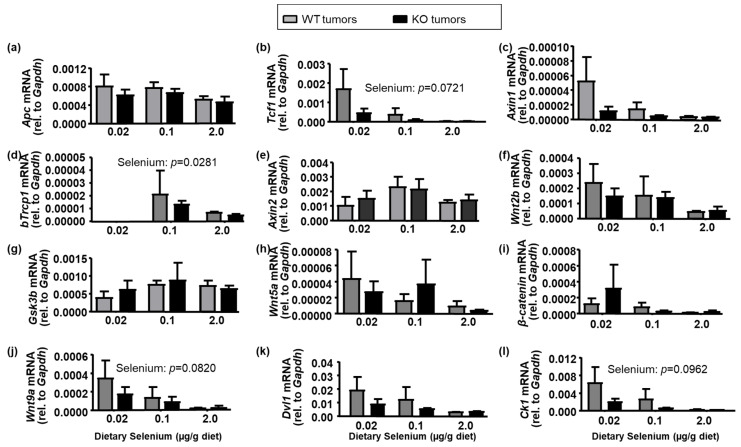
Wnt/β-catenin signaling pathway in colon tumors. mRNA was isolated from colorectal tumors of AOM/DSS-treated mice, reverse-transcribed to cDNA, and quantitatively assessed with gene-specific primers for (**a**) adenomatous poliposis coli (*Apc*), (**b**) T cell factor 1 (*Tcf1*), (**c**) *Axin1*, (**d**) β-transducin repeat containing E3 ubiquitin protein ligase pseudogene 1 (*bTrcp1*), (**e**) Axin 2, (**f**) wingless-type MMTV integration site 2b (*Wnt2b*), (**g**) glycogen synthase kinase 3β (*Gsk3b*), (**h**) *Wnt5a*, (**i**) β-catenin, (**j**) *Wnt9a*, (**k**) human homolog of the Drosophila dishevelled gene (*Dvl1*), and (**l**) casein kinase I (*Ck1*). mRNA levels for *bTrcp1* in selenium-deficient mice were below limits of detection. Means are presented (N = 4 per bar) with SEM, and were analyzed with two-way ANOVA, followed by Tukey’s *post hoc* comparisons.

The primary signaling pathway of interest in colorectal cancer development is the canonical Wnt/β-catenin signaling pathway. We quantitatively assessed mRNA expression of the Wnt/β-catenin complex in colon tumors (Figure 3) to assess whether differences in regulation of cell proliferation, invasion, and metastatic potential in colon tumors excised from both WT and Selenof-KO mice could be detected. This included adenomatous polyposis coli (*Apc*), axin1/2, glycogen synthase kinase 3β (*Gsk3β*), casein kinase 1 (*CK1*), β-transducin repeat containing gene (*bTrCP*1), the dishevelled segment polarity protein 1 (*Dvl1*), and the transcription factor T cell factor 1 (*Tcf1*). Systemic expression of *Selenof* had little to no effect on mRNA expression of genes associated with Wnt-signals or the β-catenin signaling/destruction complex. Dietary selenium exerted a very modest effect (*p* > 0.05), with expression of several genes in the Wnt/β-catenin signaling pathway suggesting a negative correlation with dietary selenium.

Additionally, we quantitated the expression of downstream targets of the Wnt/β-catenin signaling pathway in tumor tissues. Whereas *Selenof* genotype did not appear to significantly impact mRNA expression of these downstream targets (Figure S6), dietary selenium did in many cases. This was especially evident in the matrix metalloproteinases (*Mmp*) *7* and *Mmp9*, cyclin D1 (*Ccnd1*), cyclooxygenase 2 (*Cox2*), and the Jun proto-oncogene (*Jun*). Here, insufficient selenium levels resulted in a higher mRNA expression of downstream targets in tumors of AOM/DSS-treated animals.

Several other signaling pathways and molecules are known to directly or indirectly interact with the Wnt signaling pathway. This includes the Nicotinamide adenine dinucleotide phosphate (NADPH) oxidases (*Nox*) and Notch, as well as lysyl oxidase (*Lox*), and Collagen type Iα1 (*Col1a1*). The NOX are transmembrane proteins with diverse physiological functions, including playing roles in cell proliferation [39]. Notch is not only known to activate the WNT/β-catenin signaling pathway, but also interacts with NF-κB, TGFβ and Stat3, which themselves have also been shown to interact with WNT/β-catenin signaling. LOX has been implicated in the inhibition of β-catenin signaling in some cancers [40], and COL1A1 appears upregulated in colorectal cancer tissues and promotes metastasis via Wnt signaling [41]. We therefore assessed mRNA expression of these genes in tumor tissues of AOM/DSS-treated WT and Selenof-KO mice (Figure S7). mRNA expression of *Notch1* modestly correlated negatively with dietary selenium levels (*p* = 0.0655), but no statistically significant differences were observed between tumors of WT or Selenof-KO mice. Similarly, differences between WT or Selenof-KO mice were absent for *Notch2*, *Nox1*, *Stat3*, nuclear factor κ-light-chain-enhancer of activated B cells (*NF-κB*), and transforming growth factor β (*Tgfβ*,). *Col1a1* showed a slight increase in Selenof-KO tumors under selenium-deficient conditions (Figure S7), though it failed to reach statistical significance. Overall, we were unable to detect strong differences between Selenof-KO mice and WT controls in canonical signaling pathways relevant to colon carcinogenesis that would possibly have helped explain the dichotomy between ACF and tumor formation in Selenof-KO mice.

### 2.6. Intestinal Barrier Integrity

**Figure 4:**
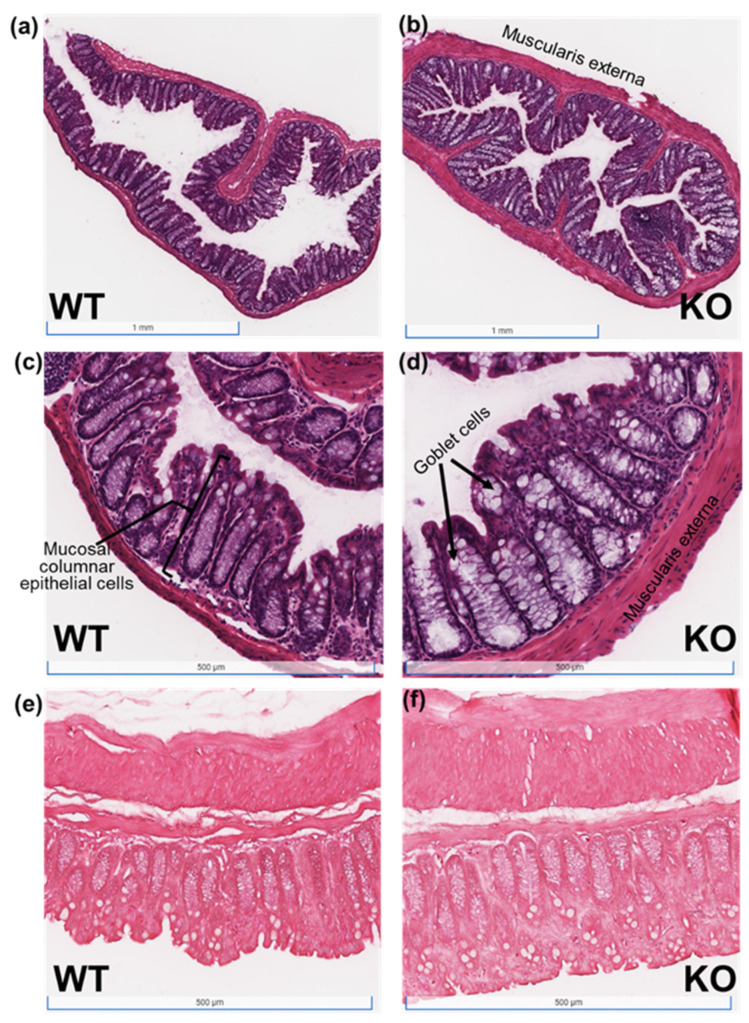
H&E and Masson’s Trichrome stains of colon tissues of WT and Selenof-KO animals. Tissue sections of untreated (control) WT and Selenof-KO animals maintained at adequate selenium levels were prepared with (**a**–**d**) hematoxylin and eosin (H&E) or (**e**,**f**) Masson’s Trichrome stains.

Given the very modest changes in expression of the investigated genes and regulatory pathways typically associated with colorectal cancer, we were interested in determining whether Selenof-KO mice exhibited differences in their mucosal morphology and expression of proteins important to barrier integrity instead. Both cross-sectional and longitudinal colon tissue sections of control WT and Selenof-KO animals maintained on adequate selenium diets were prepared with hematoxylin and eosin (H&E, Figure 4a–d) and Masson’s Trichrome stains (Figure 4e,f). Although the *muscularis externa* appeared thicker in Selenof-KO mice (Figure 4b,d,f), differences in immune cell infiltration or collagen deposition or fibrosis were not apparent in these samples. However, especially noticeable was the dramatic increase in the size of goblet cells in Selenof-KO mice (Figure 4b,d), suggesting a structural change resulting in ability of increased glycoprotein production for the mucus layer in the intestinal tract.

**Figure 5:**
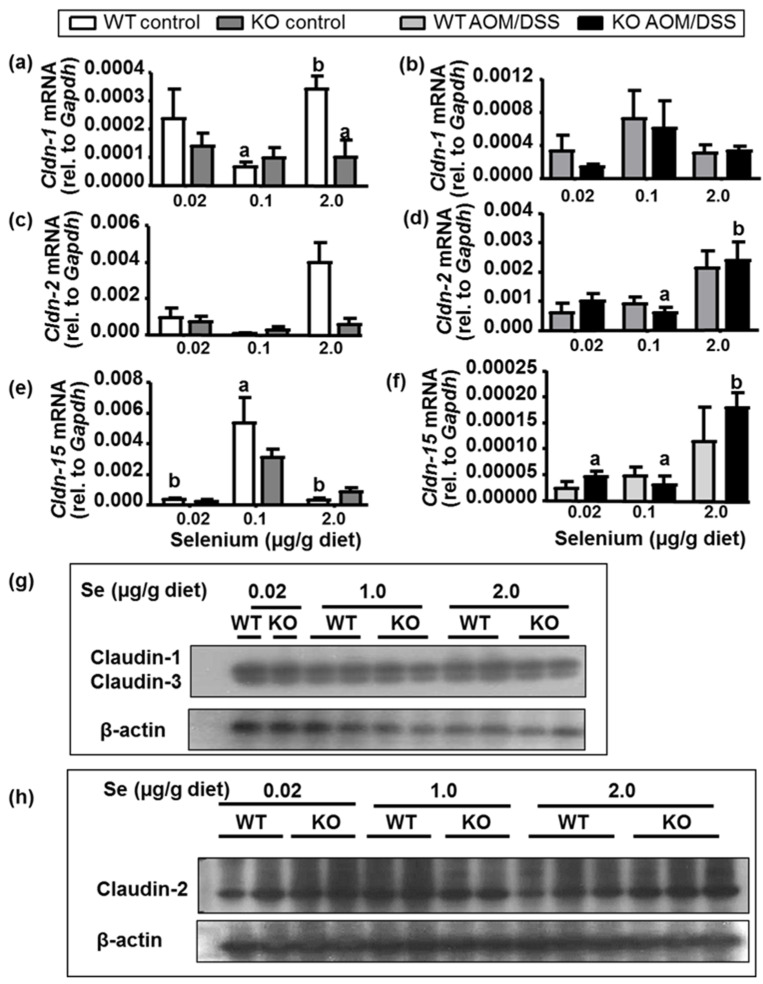
Expression of tight junction Claudin genes. mRNA expression was measured with qPCR in (**a**,**c**,**e**) colon scrapes of control mice and (**b**,**d**,**f**) colon tumors of AOM/DSS-treated mice. Mean (N = 4) + SEM, 2-way ANOVA, followed by Tukey’s post hoc analyses to compare KO vs. WT by diet; letters indicate statistically significant differences. Protein expression of (**g**) Claudin-1 and Claudin-3, and (**h**) Claudin-2 in colon scrapes of control mice on selenium-specific diets was assessed with Western blotting, using β-actin as loading control. N = 1–3 per group, 40 µg protein per lane.

We furthermore investigated the expression of tight junction and other genes known to contribute to intestinal epithelial barrier integrity in colon scrapes of untreated mice, colon tumors of AOM/DSS-treated mice (Figure 5). We did observe a significantly decreased Claudin-1 (*Cldn-1*) mRNA expression in SelenoF-KO mice under high selenium conditions in untreated animals (Figure 5a), a trend that was also seen for Claudin-2 expression (Figure 5d, *p* > 0.05). However, overall, in our in vivo model, the *Selenof* genotype showed little to no effect on mRNA expression of tight junction proteins Claudin-1 (*Cldn-1*), 2 (*Cldn-2*) and 15 (*Cldn-15*). Western blot analyses showed low expression of claudin-2 overall, and no visible differences in protein expression for Claudin-1 or Claudin-3 (Figure 5g) or Claudin-2 (Figure 5h) between WT and KO mice. It should be noted that mRNA expression of these tight junction genes in AOM/DSS-treated animals, interestingly, showed a positive correlation with dietary selenium, with significant impact on expression of *Cldn*-2 (*p* = 0.0016) and *Cldn-15* (*p* = 0.0008).

In addition to tight junction genes, we also evaluated the mRNA expression of genes typically associated with adherens junctions and other barrier integrity functions in control animals’ colon scrapes and in colon tumor tissues (Figure S8). Dietary selenium levels appeared to affect mRNA expression of the transmembrane glycoprotein epithelial cell adhesion molecule (*EpCAM*), Nectin cell adhesion molecule (*Nectin*)-2, membrane-associated carbonic anhydrase 4 (*Car4*), and the secreted glycoprotein mucin 2 (*Muc2*) in either WT or KO mice, or both. Interestingly, *Selenof*-genotype did not seem to significantly affect mRNA expression of the investigated genes in colons of mice, except for *Epcam*, which was significantly lower in tumors of Selenof-KO mice compared to WT mice, but only at high selenium levels. However, though gene expression of tight junction and adherens junction genes were not significantly altered between Selenof-KO mice and their WT littermates, the dramatically increased size of goblet cells in KO mice suggest structural changes relevant to colon tumorigenesis.

## 3. Discussion

Previous studies have suggested that the 15 kDa selenoprotein (*Selenof*) is involved in oxidative protein folding, signaling in the cellular misfolded protein response, and may function as a redox quality control for immunoglobulins [15,19,20,21,22,23]. Because functional polymorphisms of *SELENOF* exist in human populations, and have been linked to cancer outcomes, the function of this gene/protein is of great interest. Our previous in vitro and in vivo studies using colon cancer cells with a targeted down-regulation of *SelenoF* or using a systemic Selenof-KO mouse model, saw a reversal of the colon cancer phenotype [18,26] and a decreased number of chemically induced pre-neoplastic lesions [26], respectively. Herein, we evaluated the ability of Selenof-KO mice to develop tumors in an inflammatory colon tumorigenesis model, the impact of dietary selenium, and signaling mechanisms important to colorectal cancer.

Selenof-KO mice and their WT littermate controls were maintained on a selenium-deficient, selenium-adequate, or high selenium diet, were injected with the colon-specific carcinogen AOM, and exposed to the inflammatory tumor promoting agent, DSS. As expected, based on our previous in vivo study, Selenof-KO mice developed dramatically fewer ACF than WT mice, regardless of dietary selenium levels provided. Surprisingly, whereas roughly 60% of the WT mice as expected developed visible tumors in their colons, a similar percentage of Selenof-KO mice also developed tumors. This prompted our investigations to elucidate potential differences in carcinogen metabolism, cell signaling mechanisms relevant to tumorigenesis, and barrier functions specific to intestinal homeostasis.

AOM is well-known to be metabolized by the hepatic CYPE1 machinery. The resulting AOM bioactivation enables this chemical to subsequently form guanine adducts in the colon. Therefore, it was reasonable to hypothesize that Selenof-KO mice may have a lower AOM-bioactivating mechanism, and would be forming fewer ACF, but those fewer ACF might more readily develop into raised polyps and tumors. However, based on our assessment of hepatic CYP2E1 catalytic activity of the enzyme, in addition to the mRNA expression of *Cyp2e1* and other metabolizing enzymes involved in the AOM bioactivation, such a difference that would explain this phenomenon was not detected. Therefore, it appears that overall, the ability to metabolize AOM via the hepatic CYP2E1 pathway only minimally differs between mice with and without functional SELENOF, with an interesting effect of dietary selenium observed. The question remains, why and how Selenof-KO mice with very few ACF would develop a similar number and mass of tumors as WT mice with many more ACF under conditions of inflammation. It should be noted that, while ACF have often been used as biomarkers for intestinal tumorigenesis, some ACF have been shown to regress over time, and most dysplastic ACF do not progress to adenomas [42]. It is furthermore possible that Selenof-KO mice and WT mice use two different mechanisms, develop different types of colorectal tumors, and that ACF are not a good predictor for tumorigenesis in organisms with low or lacking *Selenof* expression. Thus, we continued to investigate potential mechanisms explaining this phenomenon.

Our previous study suggested that Selenof-KO mice had an altered basal inflammation status. We similarly observed in this current study a significantly higher spleen/body mass ratio in KO mice, which was exacerbated in mice exposed to AOM/DSS. We therefore assessed levels of circulating serum cytokines. However, while some serum cytokines, such as IL-6 in AOM/DSS-treated animals, were indeed significantly different in Selenof-KO mice compared to WT littermates, the fact that IL-6 can function both in an inflammatory and anti-inflammatory fashion makes interpretations difficult. We therefore focused on colon cancer-specific signaling pathways, and other factors known to impact intestinal homeostasis and therefore colon cancer.

The primary signaling pathway of interest in colorectal cancer development is the canonical Wnt/β-catenin signaling pathway. Wnt signals can activate gene transcription through nuclear localization of cytosolic β-catenin. Cytosolic levels of β-catenin are controlled by the β-catenin-destruction complex, a multimeric assembly which has been well described [43,44,45]. Loss of function mutations in the tumor suppressor adenomatous polyposis coli protein (APC) pre-disposes to colorectal adenomas and colorectal cancer, and the vast majority of sporadic colon tumors are found to have mutations in APC [45,46,47,48,49,50]. Stimulation with the Wnt signal typically leads to the nuclear translocation of β-catenin, and through β-catenin binding to transcriptional activators subsequently to expression of genes important in cell proliferation (e.g., cMYC, cyclins) and cell migration (e.g., matrix metalloproteases, e-cadherin, vascular endothelial growth factor (VEGF)). In addition to its nuclear role in regulating cell proliferation via its downstream targets, membrane-associated stable β-catenin is also involved in regulation and coordination of cell–cell adhesion through its responsibility of the anchoring of cadherins as part of mammalian cell adhesion complexes [51,52], thus impacting barrier integrity in intestinal tissues. Additionally, during colon tumorigenesis, the morphology and synthesis of collagen fibers and other proteins present or active in the extracellular matrix are known to change. Expression of matrix metalloproteinases (MMPs) 2 and MMP9, as well as lysyl oxidase (LOX) are deemed important contributors to tumor invasion and metastasis [53], and are linked to the Wnt/β-catenin pathway. *Selenof* expression did not significantly impact mRNA expression of components of the Wnt/β-catenin pathway or of its downstream targets in colon tumors of mice. Interestingly, dietary selenium modulated mRNA expression of various targets, especially in colon tumors. In mice on deficient selenium levels, a higher mRNA expression of downstream targets was observed in tumors of AOM/DSS-treated animals, which correlated with greater tumor mass. This may, at least in part, explain observations in animal models, where selenium deficiency has been shown to affect the Wnt/β-catenin pathway [54], and epidemiological studies, where selenium status inversely correlated with both cancer incidence and mortality [8].

Several other pathways are known to interact with the Wnt/β-catenin signaling pathway. This includes the NADPH oxidases (NOX), which play roles in cell proliferation via generating ROS [39], and the Notch family of receptors that plays a role in tissue homeostasis and metabolism [55] and in regulation of stem cell properties and cell differentiation [56,57]. Furthermore, the signal transducer and activator of transcription (Stat)-3 is a downstream signaling molecule of IL-6, and acts as a transcription factor with various signaling pathways, including Notch, Nox, and Wnt/β-catenin. Lysyl oxidases (LOX), on the other hand, have been implicated in the inhibition of β-catenin signaling in some cancers [40], whereas the COL1A1 protein appears upregulated in colorectal cancer tissues and promotes metastasis via Wnt signaling [41]. Again, we found some interesting trends where dietary selenium seems to negatively correlate with *Notch* and *Nox* expression, potentially explaining, in part, how selenium deficiency may contribute to increased tumorigenesis. However, much like was observed for the Wnt/β-catenin signaling pathway, neither *Lox*, *Col1a1*, *Tgfβ*, nor *NF-κB* mRNA levels were significantly impacted by *Selenof*-genotype in tumor tissues of AOM/DSS-treated WT and Selenof-KO mice.

Therefore, with the major signaling pathways linked to colorectal tumorigenesis unlikely being significantly modulated by *Selenof* expression, we shifted our focus to the intestinal barrier homeostasis. The single cell layer that forms the intestinal epithelial barrier, is held together by various intercellular junctions that control and regulate permeability and homeostasis in the intestinal epithelium via adherens junctions, desmosomes, and apical tight junctions. Multiple important pathways, including Wnt/β-catenin and Notch/Nox signaling pathways, intersect with the regulation or expression of proteins important in regulation of the intestinal epithelial barrier. Among the many barrier proteins, the members of the families of claudins and occludin localize at and are major constituents of tight junction complexes [58]. Occludin is a cytokine-regulated integral membrane protein that induces adhesion [59], and claudins are involved in selectively controlling paracellular movement of ions [60]. Furthermore, the expression of *CLAUDIN-1* and *CLAUDIN-2* appears elevated in inflammatory bowel diseases and is thought to contribute to tumor progression [61,62]. We hypothesized that mice lacking *Selenof* expressed barrier integrity proteins in intestinal tissues differently than their WT littermate controls, which would result in altered expression of enzymes important to remodeling of colon mucosa and submucosa. This, in turn, could potentially impact the response to a colon carcinogen and/or an inflammatory agent, and possibly explain why Selenof-KO mice develop tumors albeit lacking the development of persistent ACF. In our model, mRNA expression of tight junction proteins claudin-1 and -2, appeared substantially lower in Selenof-KO mice under high-selenium conditions. While such a decrease in claudin-2 would suggest a higher barrier integrity, these observed decreases were not found in tumor tissues from these animals. Furthermore, no differences were found for protein expression for tight junction proteins claudin-1 or -2 in mice. Similarly, mRNA expression of genes relating to adherens junctions, such as the transmembrane glycoprotein epithelial cell adhesion molecule (*Epcam*) which contributes to intercellular adhesion [63], or those relating to general barrier integrity showed interesting trends based on dietary selenium, but not based on *Selenof*-expression of the mice.

However, supporting evidence of altered homeostasis in barrier integrity was observed in tissue sections of untreated (control) WT and Selenof-KO animals, which we prepared with hematoxylin and eosin (H&E) or Masson trichrome stains. Given the possible increased basal systemic inflammation as evidenced by frequent splenomegaly in Selenof-KO mice, we anticipated indications of increased fibrosis, but that was not detected in any of the tissues examined. Instead, the most striking difference was the enormous increase in the size of goblet cells in Selenof-KO mice, though it’s unclear whether this was with a concomitant increase in number of goblet cells. Regardless, this finding suggests the potential for increased mucin production in Selenof-KO animals. Mucins are high molecular weight transmembrane glycoproteins that are produced by goblet cells in colonic epithelia, and have been shown to be over-expressed in various cancers, including colorectal cancer. Among these mucins, Mucin-2 (*MUC-2*) is the major secreted form, shown to be expressed by intestinal adenomas and especially by mucinous carcinomas [64,65]. In vivo studies demonstrated that high mucin variant cells injected into nude mice formed twice as large tumors as those of parental cells [65]. In parallel, patients with mucin-producing colorectal cancer appear to have a poor prognosis in terms of outcome. In our study, though we had expected to be able to detect an increase in *Muc-2* transcription in Selenof-KO mice, given the observed increase in goblet cell size, no increase in *Muc-2* mRNA was detected in scrapes of colons nor in colon polyps. We recognize that because colon scrapes constitute a mixture of cell types, it is possible that any changes in gene expression of goblet cells were masked by those of other cell types in the samples. Based on our findings with increased goblet cell sizes in Selenof-KO mice, we furthermore acknowledge the potential role of intestinal microbiota that might contribute to or be the result of potential differences in mucus composition, and therefore impact intestinal barrier integrity. While this is currently beyond the scope of this manuscript, we are looking forward to investigating this in future studies.

Our previous studies suggested that CT-26 mouse colon cancer cells lacking SELENOF displayed limitations in terms of invasion, metastasis, and cell replication [17]. However, these mouse colon cancer cells, though being adenocarcinoma cells, are not considered mucinous carcinoma cells. Mucinous carcinoma cells generally possess a higher degree of invasiveness [66]. Therefore, colorectal cancer cells that are derived from adenocarcinoma cells with low *Selenof* expression may be less aggressive or invasive as we had observed in vitro [17,18]. Whether mucinous carcinoma cells would respond similarly to changes in Selenof-expression remains to be elucidated. Because it has been shown that the predominant mechanisms of tumor progression differ between mucinous carcinoma cells and colorectal adenocarcinoma cells [66], these differences in cell types from which tumors and pre-neoplastic lesions can develop, may explain why Selenof-KO mice appear to be protected initially against ACF formation, but not AOM/DSS-induced tumorigenesis. Therefore, our study showed that Selenof-KO developed tumors in an AOM/DSS-model of colon carcinogenesis, albeit forming dramatically fewer aberrant crypt foci than observed in WT animals. Our main findings showed structural changes in the intestinal tissues of Selenof-KO mice that suggest an altered intestinal barrier integrity.

## 4. Materials and Methods

### 4.1. Materials

NuPage^®^ 4–12% polyacrylamide gels, LDS sample buffer, See-Blue Plus2 protein markers, and TRIzol^®^ reagent were purchased from Invitrogen (Carlsbad, CA, USA); iScript™ cDNA synthesis Kit and SYBR™ green supermix from Bio-Rad Laboratories (Hercules, CA, USA), primers for real-time PCR from Integrated DNA Technologies (Coralville, IA, USA). Antibodies against Claudin-1 (which also recognizes Claudin-3) and -2 were purchased from ThermoFisher Scientific (Waltham, MA, USA). Goat polyclonal actin primary antibody, and horseradish peroxidase-conjugated secondary antibody were obtained from Santa Cruz Biotechnology (Santa Cruz, CA, USA), and SuperSignal West Dura substrate from Pierce (Rockford, IL, USA). A mouse TH1/TH2 9-Plex assay kit was purchased from MesoScale Discovery (Gaithersburg, MD, USA). All other reagents used were commercially available and were of the highest quality available.

### 4.2. Animal Care Disclosure and Study Organization

All mice used in this experiment were maintained at the National Cancer Institute (National Institutes of Health (NIH)) and were handled and sacrificed in a humane manner in strict accordance with the recommendations in the Guide for the Care and Use of Laboratory Animals of the NIH in Bethesda, Maryland. The Animal Ethics Committee at the NIH previously approved these experiments with proper permit documentation (LCP-011) obtained from the Institutional Animal Care and Use Committee, and documents are on file both at the NIH and at Towson University. Selenof-KO mice (KO) lacking exon 2 of the gene and thus lacking the functional SELENOF protein were generated as described previously [20], and only male Selenof-KO mice and littermate controls (WT) were used to eliminate sex as a variable. Genotypes of the animals were verified by PCR using the following primers: WT allele detection (250 bp): 59-CAGAGTTTGCGTCAGAGGCA-TGCAGAG-39 and 59-CTGAAACTCGTAAAGTCAGAGACTACTGG-39; KO allele detection (312 bp): 59-GGTGTGTTTGCAGATAAGCTAATGC-39 and 59-TACCCGGTAGAATTGACCTGCAG-39.

Weanling mice of both genotypes were weighed, and randomly assigned to be fed a Torula yeast-based customized chow with sodium selenite at 0.02 µg/g diet (selenium-deficient), 0.1 µg/g diet (selenium-adequate), or 2.0 µg/g diet (high-selenium) for the duration of the study (Figure S1). Animals were given free access to deionized water and were monitored closely for any clinical signs of poor health throughout the study. Animals were subcutaneously injected with either azoxymenthane (AOM, 10 mg/kg solubilized in ~100 µL saline) or saline only (controls) at six weeks of age, after having been fed their respective selenium-specific diets for three weeks. At seven weeks of age, AOM-injected mice were given two one-week treatments with 2% dextran sulfate sodium (DSS) via their drinking water separated by a one-week recovery period. All mice were weighed twice weekly for the first 10 weeks in the study, and every other week thereafter. At ten weeks, all mice were maintained on regular drinking water alongside their respective customized selenium diet until the end of the study at 20 weeks (Figure S1). Mice were sacrificed using CO_2_ asphyxiation. Animals were weighed, tissues (after determining organ weights) and serum were harvested, flash frozen, and stored at −80 °C for subsequent analyses.

### 4.3. Colorectal Tumor and ACF Analyses

Colons from all animals were excised from anus to caecum and rinsed with sterile Dulbecco’s phosphate-buffered saline (DPBS). Each colon was measured from anus to caecum in centimeters with a ruler, accurate to one millimeter, opened longitudinally, and stored in 70% ethanol or 10% formalin for subsequent analysis, unless the tissue was used for gene expression analysis. Tumor formation was measured by two independent examiners, counting the total number of tumors formed in each colon using a dissecting microscope. A select number of tumors were excised prior to tissue fixation, the mass of each tumor was determined using a digital scale accurate to 10^−4^ g, and flash frozen for gene expression analyses. To quantitate formation of ACF, ethanol-stored colonic tissues were stained with methylene blue (1 g/L in DPBS) and examined using a dissecting microscope by an examiner blinded to the animal’s genotype or treatment to avoid any detection bias. The means were calculated for tumor number, tumor mass, and number of ACF formed in each genotype and treatment group.

### 4.4. Tissue Staining

Colon tissues of untreated animals were embedded into paraffin and sectioned with a microtome and fixed to glass slides. Subsequently, sections were dewaxed with xylene, washed with ethanol, rinsed with water, and stained with either haemotoxylin and eosin (H&E) to identify acidic structures like nuclei blue and basic structures such as cytoplasm pink, or Masson’s Trichrome (MTC) to stain cytoplasm and muscle fibers red, and collagen with aniline blue. Slides were scanned using Johns Hopkins Medical Institute’s Oncology Tissue Services, and images were evaluated by three independent observers.

### 4.5. Gene Expression Analysis of Mouse Liver and Colon Tissues

For subsequent real-time RT-PCR, total RNA was isolated from liver and colon tissues using the TRIzol (Thermo Fisher Scientific, Carlsbad, CA, USA) reagent following the manufacturer’s recommendation, and reverse transcribed using the iScript cDNA synthesis kit (BioRad, Herkules, CA, USA) with 1 µg of total RNA. Gene expression was assessed via real-time RT-PCR using iTaq Universal SYBR Green Supermix (BioRad, Herkules, CA, USA) according to the manufacturer’s instructions in 10 µL reactions. mRNA expression was normalized to the expression of glyceraldehyde-3-phosphate dehydrogenase (*Gapdh*).

For Western blotting analyses, colon scrapes were homogenized in lysis buffer with protease inhibitors. Extracted cell lysates were prepared for denaturing gel electrophoresis using NuPAGE LDS 4x sample buffer, heated at 70 °C for 10 min, and 40 µg protein/lane were electrophoresed on NuPAGE 4–12% Bis-Tris polyacrylamide gels. Subsequently, proteins were transferred to polyvinylidene difluoride membranes, and the membranes were blocked in 1% bovine serum albumin in Tris-buffered saline with 0.1% Tween 20 (TBST) for a minimum of 1 h. Membranes were incubated with primary antibodies against Claudin-1 or Claudin-2 for a minimum of one h (1:1000), and then washed in TBST for 10 min three times. Horseradish peroxidase-conjugated secondary antibody (1:10,000) was applied for two h, and the membranes were incubated in Pierce chemiluminescent substrate (ThermoFisher Scientific, Carlsbad, CA, USA) and exposed to X-ray film for detection.

### 4.6. Cyp2e1 Catalytic Activity Assay

Liver microsomes were isolated following Schenkman and Cinti’s protocol [67]. Briefly, liver tissues were homogenized in 0.25 M sucrose in 10 mM Tris-chloride (pH 7.4) and centrifuged at 12,000× *g*. CaCl_2_ (8.0 mM final concentration) was added, and microsomes were pelleted via centrifugation at 25,000× *g* for 15 min and resuspended in 50–75 µL 10 mM KPi/125 mM KCl buffer. CYP2E1 enzyme activity was measured after the modified protocol of Cederbaum [68], using 0.2–0.5 mg microsomal protein and para-nitrophenol to detect formation of para-nitrocatechol at 37 °C. Reactions were initiated by addition of NADPH (1 mM final concentration), and terminated after 10 min by adding trichloroacetic acid (1% final concentration), as described [68]. Proteins were precipitated via centrifugation, and absorbance at 510 nm of the NaOH-treated supernatant was determined with a VersaMax spectrophotometer (ThermoFisher Scientific, Waltham, MA, USA). Para-nitrocatechol concentrations were determined from the extinction coefficient 9.53 mM^−1^ cm^−1^.

### 4.7. Serum Cytokine Analysis

Blood was collected from mice by cardiac puncture at sacrifice and centrifuged in heparinized tubes at 3000× *g* for five min. Serum was then frozen and stored at −80 °C until further analysis. Using the mouse TH1/TH2 7-Plex assay kit, protein levels of interferon-γ, interleukin (IL)-12p70, IL-6, tumor necrosis factor (TNF)-α, KC/GRO (CXCL1, GROα,), IL-1β, and IL-10 were measured in a sandwich immunoassay format using a SECTOR Imager 2400 per manufacturer’s protocol (MesoScale Discovery, Rockville, MD, USA). An eight-point standard curve was used to calculate the concentration of cytokines in each murine serum sample, and all samples and standards were analyzed in duplicate (technical replicates).

### 4.8. Epigenetic Analyses

Genomic DNA was isolated from liver tissues using FitAmp DNA extraction kits (Epigentek, Farmingdale, NY, USA), and global 5-mC DNA methylation was detected using a MethylFlash colorimetric methylated DNA quantification kit (EpiGentek, Farmingdale, NY, USA) following the manufacturer’s protocols, with the percentage of methylated DNA proportional to the optical intensity measured with the VersaMax plate reader. Nuclear extracts from mouse livers were isolated using the EpiQuik Nuclear Extraction Kit (EpiGentek, Farmingdale, NY, USA).

### 4.9. Statistical Analyses

Unless otherwise indicated, data are presented as means +/− SEM, and group means were analyzed with one-way or two-way ANOVA, as appropriate, using GraphPad Prism (v. 9, GraphPad Software, San Diego, CA, USA), followed by Tukey’s post hoc analyses. Levels of significance were set to α = 0.05.

## 5. Conclusions

Systemic expression of the 15 kDa selenoprotein, *Selenof*, has been thought to impact cancers in a tissue-specific manner. Whereas effects of *Selenof*-expression in lung cancer cell lines resulted in minimal effects, the effects of *Selenof* in colorectal cancer appeared to be much more substantial [17,18,24]. However, the mechanism behind the reversal of the cancer phenotype in human and mouse colon cancer cells, as well as the dramatic reduction in chemically induced pre-neoplastic lesions in an in vivo Selenof-KO model remained unclear. Our study showed for the first time that the Selenof-KO mouse is capable of developing large tumors in an AOM/DSS-model of colon carcinogenesis albeit forming dramatically fewer aberrant crypt foci than WT animals. Given that the Selenof-KO mouse does not have a strong phenotype other than the early development of cataracts, it may not be surprising that the molecular mechanism remains elusive. Tight junction and other barrier integrity genes appear to have only minor differences in terms of expression, though we recognize the caveat of having to investigate mixtures of cell types present in colon scrapes that may mask any true differences, which will have to be further elucidated. Our main findings point to *Selenof*’s potential role in intestinal barrier integrity and structural changes in glandular and mucin-producing cells in the mucosa and submucosa. Such goblet cells are integral parts of epithelial surfaces in the intestinal barrier but also at the front of the eyes. It would be tempting to speculate that potential changes in intestinal goblet cells would indicate systemic changes that would also affect conjunctival goblet cells, which secrete soluble mucins for the ocular tear film. However, while a protective function of conjunctival goblet cells for regulating surface immune homeostasis is multi-faceted [69], dysregulation of conjunctival mucins generally does not seem to result in cataract development, which is the phenotype observed in Selenof-KO mice [20]. However, our findings of structural changes in intestinal barrier may be of interest to human health, should single nucleotide polymorphisms in the human *SELENOF* gene result in differential expression or activity of SELENOF in the colon. Whether and how this may be further modulated by dietary selenium intake continues to be an area of further studies.

## Figures and Tables

**Table 1:** Incidence of aberrant crypt foci in untreated (control) and AOM/DSS-treated mice.

Selenium (µg/g Diet)	WT Control ^1^	WT AOM/DSS ^1^	Selenof-KO Control ^1^	Selenof-KO AOM/DSS ^1^
0.02	0/8 (0%)	13/15 (86.7%)	1/9 (11.1%)	4/14 (28.6%)
0.1	2/8 (25%)	13/18 (72.2%)	0/9 (0%)	3/11 (27.3%)
2.0	2/9 (22%)	10/13 (76.9%)	0/12 (0%)	6/14 (42.9%)

^1^ number of mice with ACF/total number of mice in group (percentage).

## Data Availability

Data generated during the study are contained within the article and Supplementary Materials.

## Supplementary Materials

The Supplementary Materials are available online at https://www.mdpi.com/article/10.3390/ijms221910651/s1.
