# Supplementary material for: Selenium and the 15kDa Selenoprotein Impact Colorectal Tumorigenesis by Modulating Intestinal Barrier Integrity
Source: Int J Mol Sci. 2021 Sep 30;22(19):10651. doi: 10.3390/ijms221910651 (PMC8508755; doi:10.3390/ijms221910651)
Supplement: Supplementary file 1 [file ijms-22-10651-s001.zip › Supplementary Figures S1-S8/Figure S1.pdf]

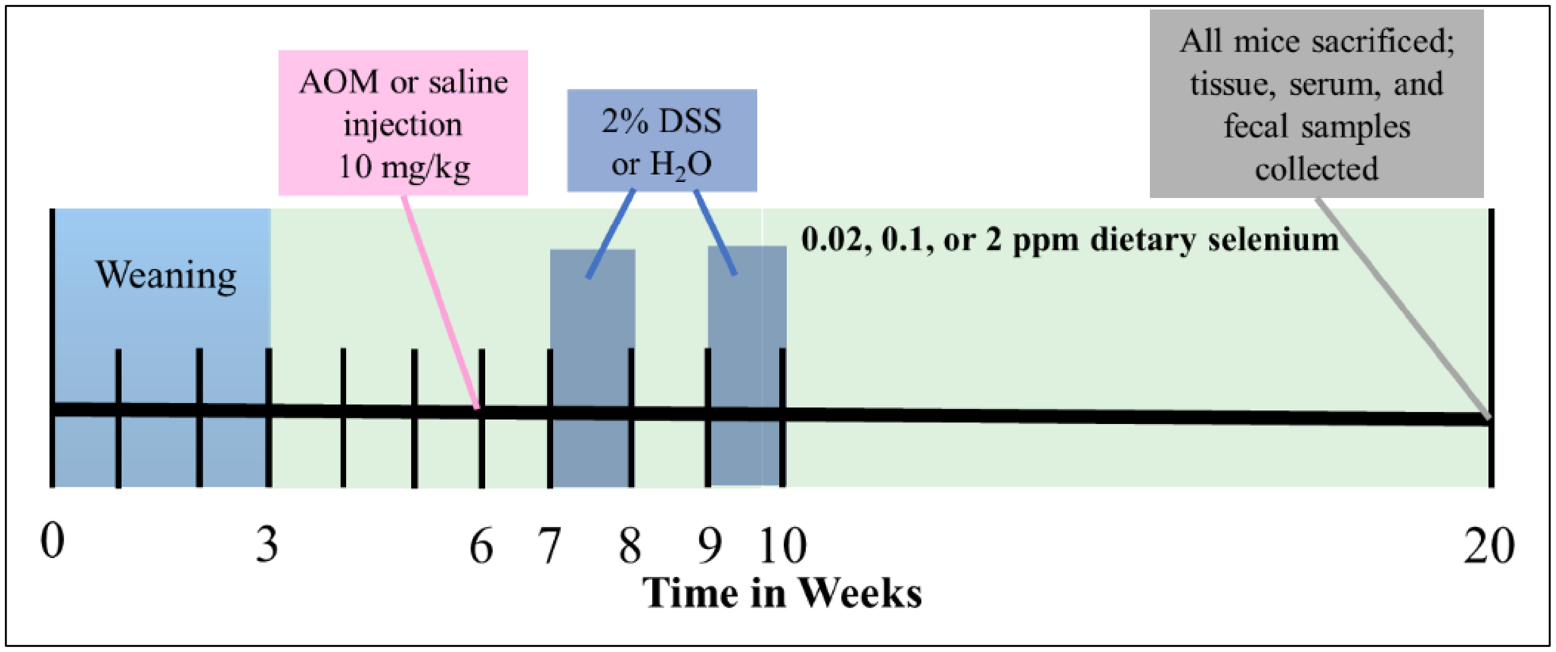

Figure S1. Experimental design of Selenof-KO and WT littermate controls in colon tumorigenesis**.** Each group included 10-12 male mice, which were kept in the same animal facility in cages with up to 5 animals, and thus exposed to the same environmental conditions. Mice were maintained on a Torula yeast-based diet with either 0.02, 0.1, or 2.0 µg sodium selenite per g diet post-weaning.
