# Supplementary material for: Selenium and the 15kDa Selenoprotein Impact Colorectal Tumorigenesis by Modulating Intestinal Barrier Integrity
Source: Int J Mol Sci. 2021 Sep 30;22(19):10651. doi: 10.3390/ijms221910651 (PMC8508755; doi:10.3390/ijms221910651)
Supplement: Supplementary file 1 [file ijms-22-10651-s001.zip › Supplementary Figures S1-S8/Figure S2.pdf]

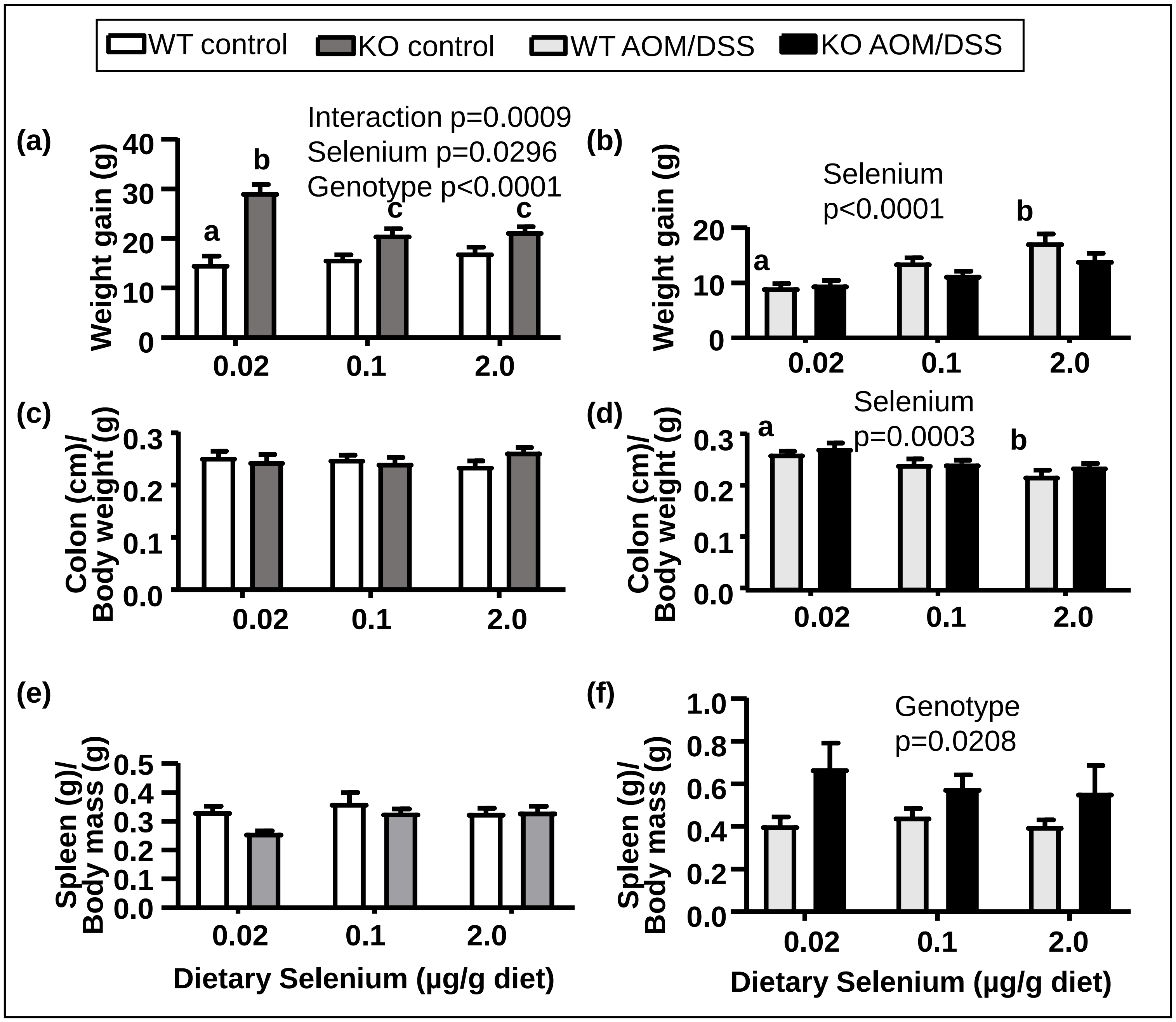

Figure S2. Growth metrics of Selenof-KO (KO) and wildtype littermate mice (WT) exposed to either AOM/DSS or saline/water (control). **(a)** Weight gain of mice over the course of the study in control and **(b)** AOM/DSS-treated animals. **(c)** Relative colon length was determined as a ratio to total body mass in control and **(d)** AOM/DSS-treated animals. Similarly, **(e)** relative spleen mass in control and **(f)** in AOM/DSS-treated animals was determined as a ratio to total body mass. Bars represent mean (N=10-12) + SEM; analyzed with two-way ANOVA, followed by Tukey's *post-hoc* comparisons.
