# Supplementary material for: Selenium and the 15kDa Selenoprotein Impact Colorectal Tumorigenesis by Modulating Intestinal Barrier Integrity
Source: Int J Mol Sci. 2021 Sep 30;22(19):10651. doi: 10.3390/ijms221910651 (PMC8508755; doi:10.3390/ijms221910651)
Supplement: Supplementary file 1 [file ijms-22-10651-s001.zip › Supplementary Figures S1-S8/Figure S3.pdf]

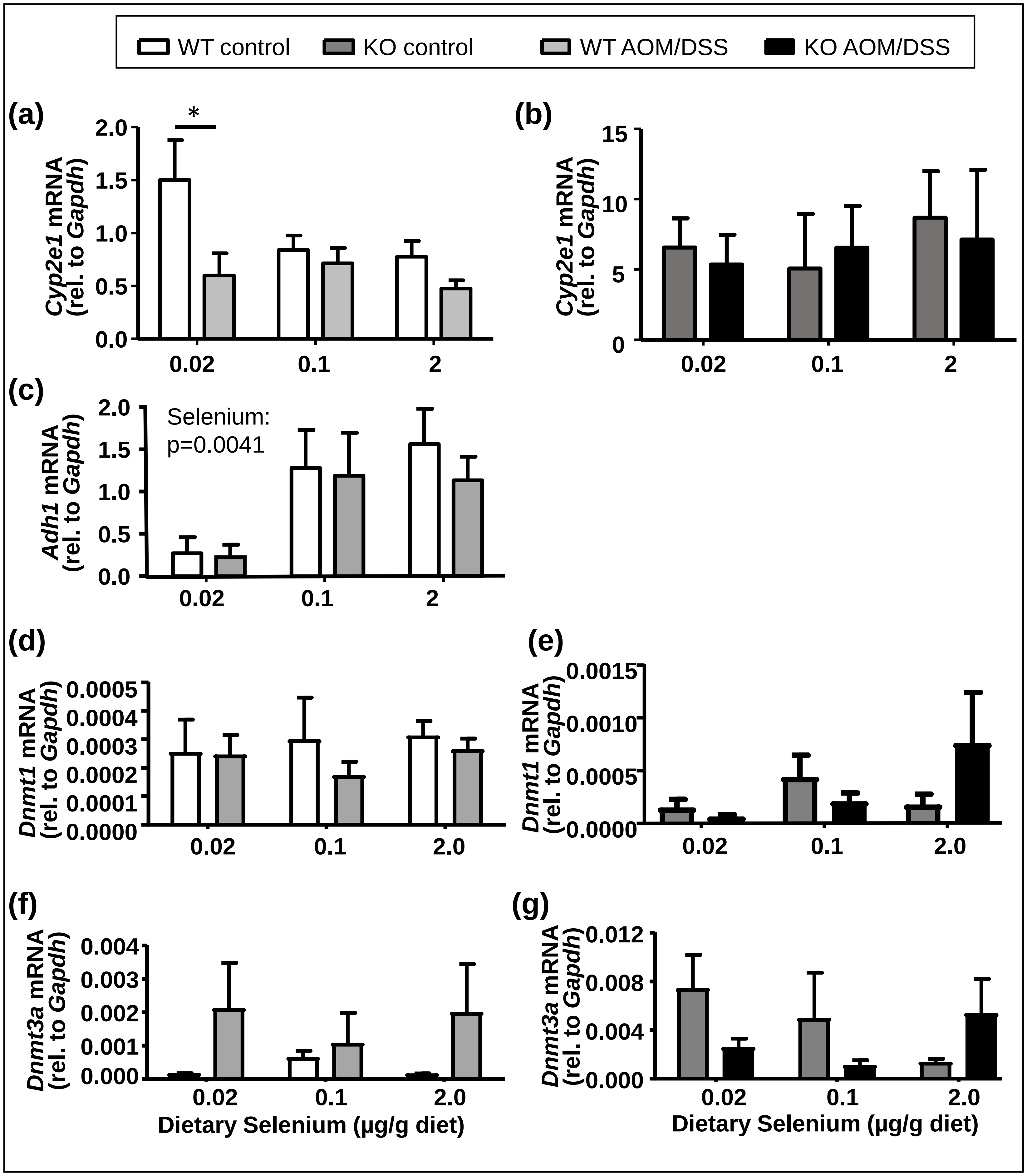

Figure S3. Hepatic mRNA Expression of AOM-metabolism enzymes. **(a)** Baseline *CYP2e1* mRNA levels were significantly elevated in WT mice on selenium-deficient diets (0.02 µg/g diet; $p<0.05$), but not in **(b)** AOM/DSS-treated animals. mRNA levels of **(c)** *Adh1*, **(d)** *Dnmt1* in control animals, **(e)** *Dnmt1* in AOM/DSS-treated mice, **(f)** *Dnmt3a* in control animals and **(g)** *Dnmt3a* in AOM/DSS-treated animals did not differ between genotypes. Bars represent means (N=8) + SEM, analyzed by 2-way ANOVA.
