# Supplementary material for: Selenium and the 15kDa Selenoprotein Impact Colorectal Tumorigenesis by Modulating Intestinal Barrier Integrity
Source: Int J Mol Sci. 2021 Sep 30;22(19):10651. doi: 10.3390/ijms221910651 (PMC8508755; doi:10.3390/ijms221910651)
Supplement: Supplementary file 1 [file ijms-22-10651-s001.zip › Supplementary Figures S1-S8/Figure S4.pdf]

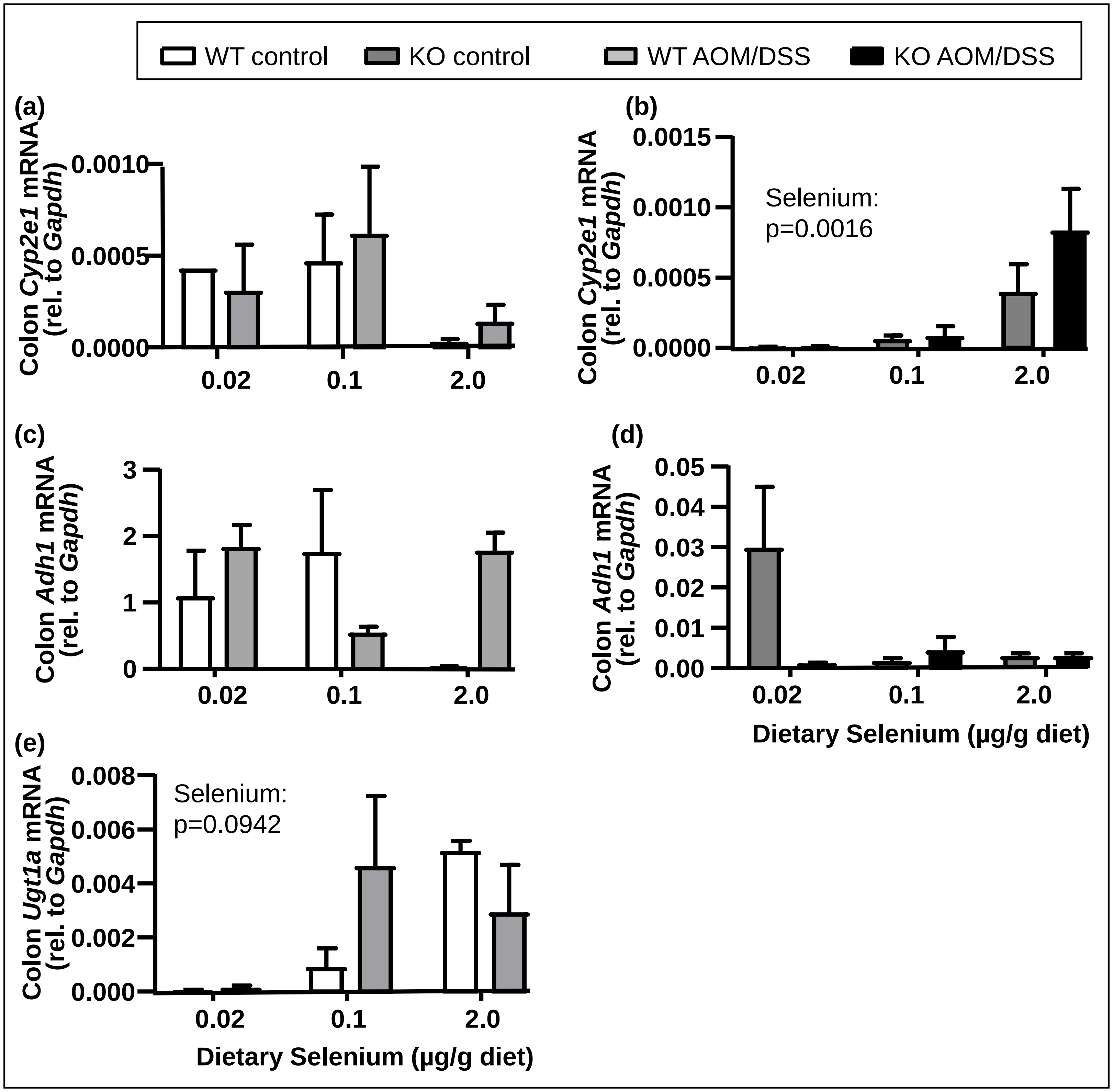

Figure S4. Colon mRNA expression of AOM-metabolism enzymes. *CYP2e1* in **(a)** control and **(b**) AOM/DSS-treated mice. *Adh1* mRNA in **(c)** control and **(d)** AOM/DSS-treated mice. **(e)** *Ugt1a* mRNA in control mice (*Ugt1a* mRNA was below limits of detection in AOM/DSS treated animals). Bars represent means (N=4) + SEM, analyzed by 2-way ANOVA.
