# Supplementary material for: Selenium and the 15kDa Selenoprotein Impact Colorectal Tumorigenesis by Modulating Intestinal Barrier Integrity
Source: Int J Mol Sci. 2021 Sep 30;22(19):10651. doi: 10.3390/ijms221910651 (PMC8508755; doi:10.3390/ijms221910651)
Supplement: Supplementary file 1 [file ijms-22-10651-s001.zip › Supplementary Figures S1-S8/Figure S5.pdf]

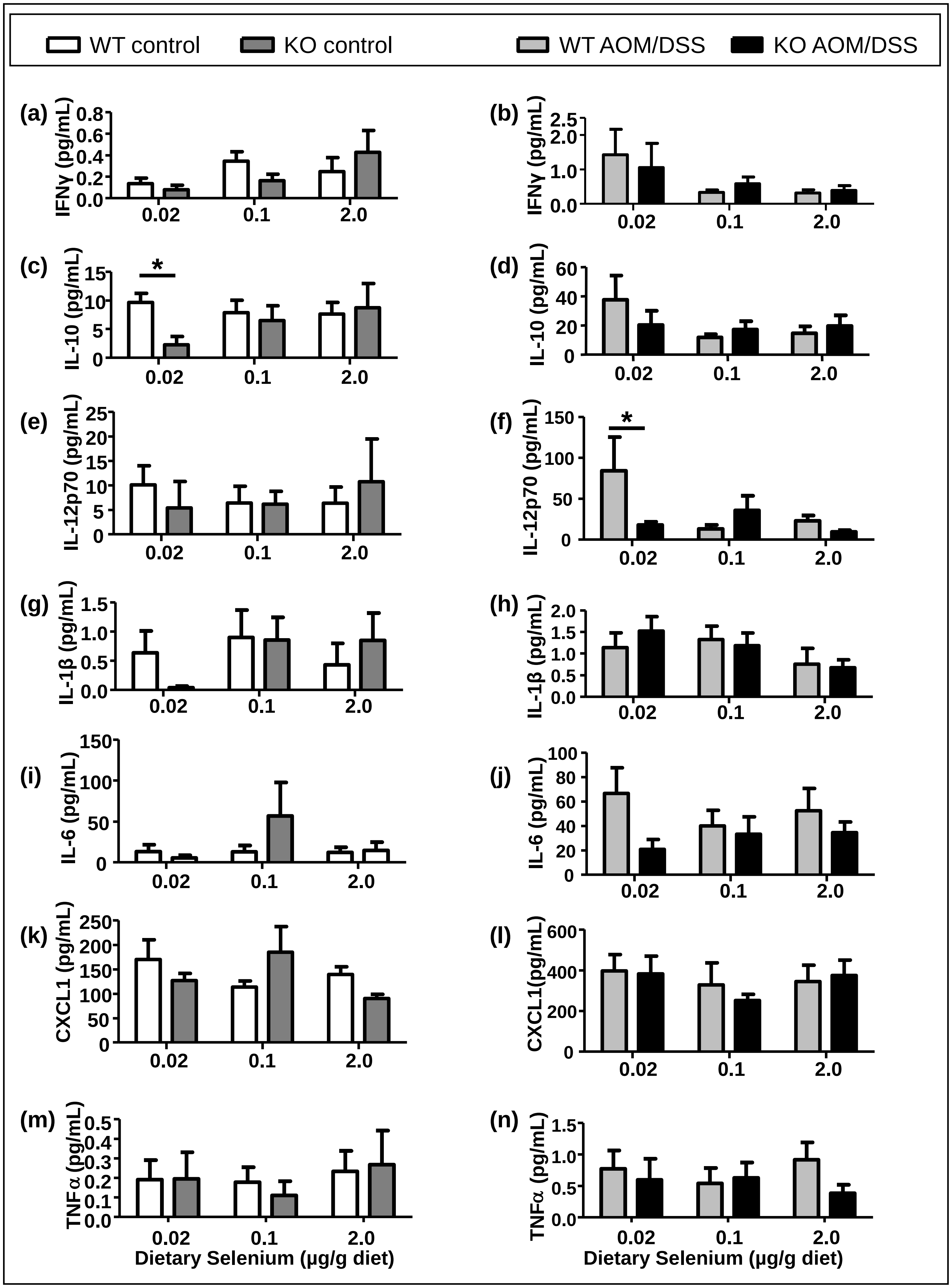

Figure S5. Serum cytokines. Circulating serum cytokines were measured in untreated and AOM/DSS-treated WT and Selenof-KO mice using the mouse TH1/TH2 7-Plex assay kit in a sandwich immunoassay format. Data were analyzed with two-way ANOVA (N=6/bar, mean + SEM). Significant differences are indicate by *p<0.05.
