# Supplementary material for: Selenium and the 15kDa Selenoprotein Impact Colorectal Tumorigenesis by Modulating Intestinal Barrier Integrity
Source: Int J Mol Sci. 2021 Sep 30;22(19):10651. doi: 10.3390/ijms221910651 (PMC8508755; doi:10.3390/ijms221910651)
Supplement: Supplementary file 1 [file ijms-22-10651-s001.zip › Supplementary Figures S1-S8/Figure S6.pdf]

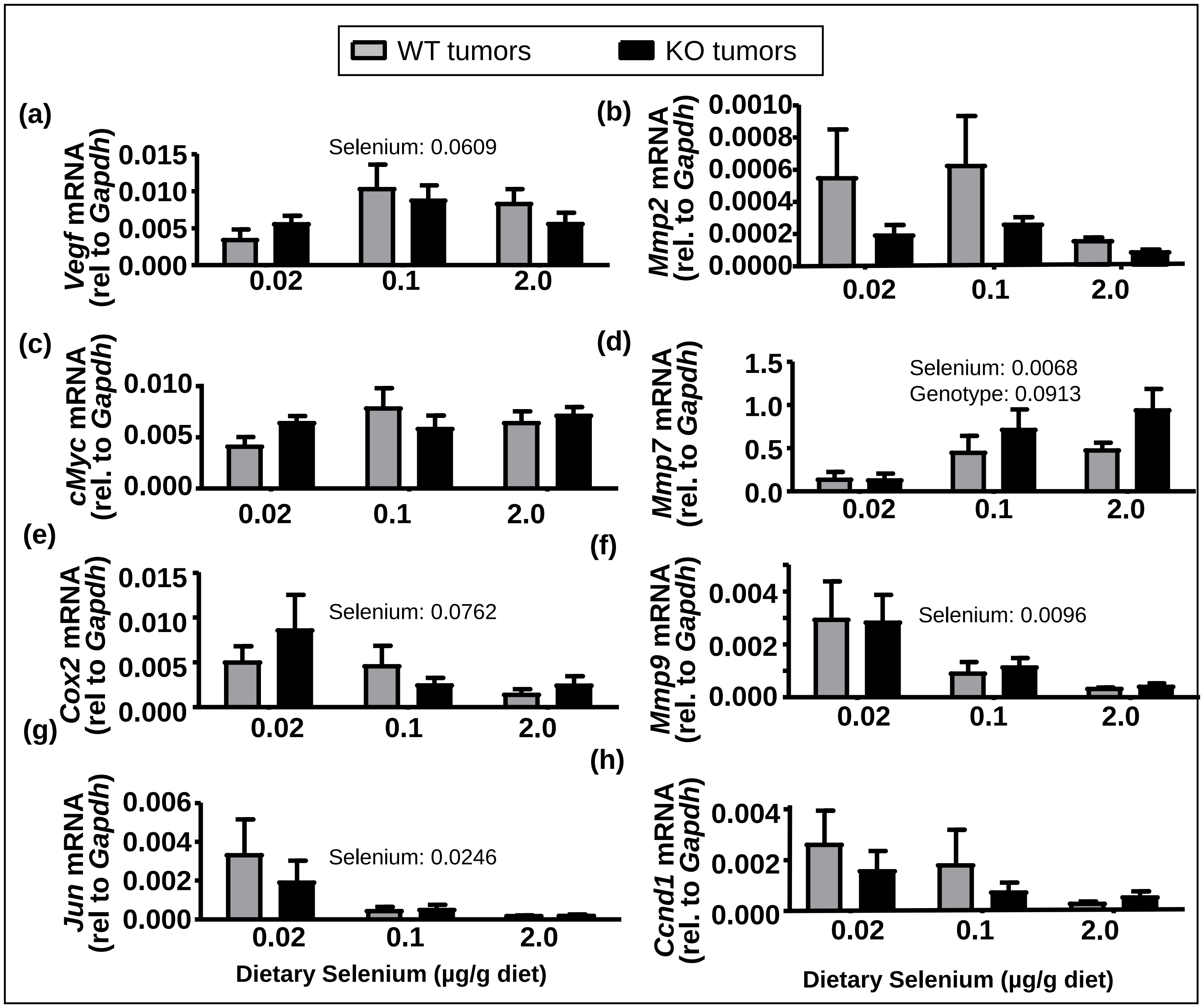

Figure S6. Downstream targets of the Wnt/β-catenin signaling pathway in colon tumors are affected by dietary selenium. mRNA was isolated from colon tumors of AOM/DSS-treated mice, reverse-transcribed to cDNA, and quantitatively assessed with gene-specific primers. Means are presented (N=4 per bar) with SEM, and were analyzed with two-way ANOVA, followed by Tukey's *post-hoc* comparisons.
