# Supplementary material for: Selenium and the 15kDa Selenoprotein Impact Colorectal Tumorigenesis by Modulating Intestinal Barrier Integrity
Source: Int J Mol Sci. 2021 Sep 30;22(19):10651. doi: 10.3390/ijms221910651 (PMC8508755; doi:10.3390/ijms221910651)
Supplement: Supplementary file 1 [file ijms-22-10651-s001.zip › Supplementary Figures S1-S8/Figure S7.pdf]

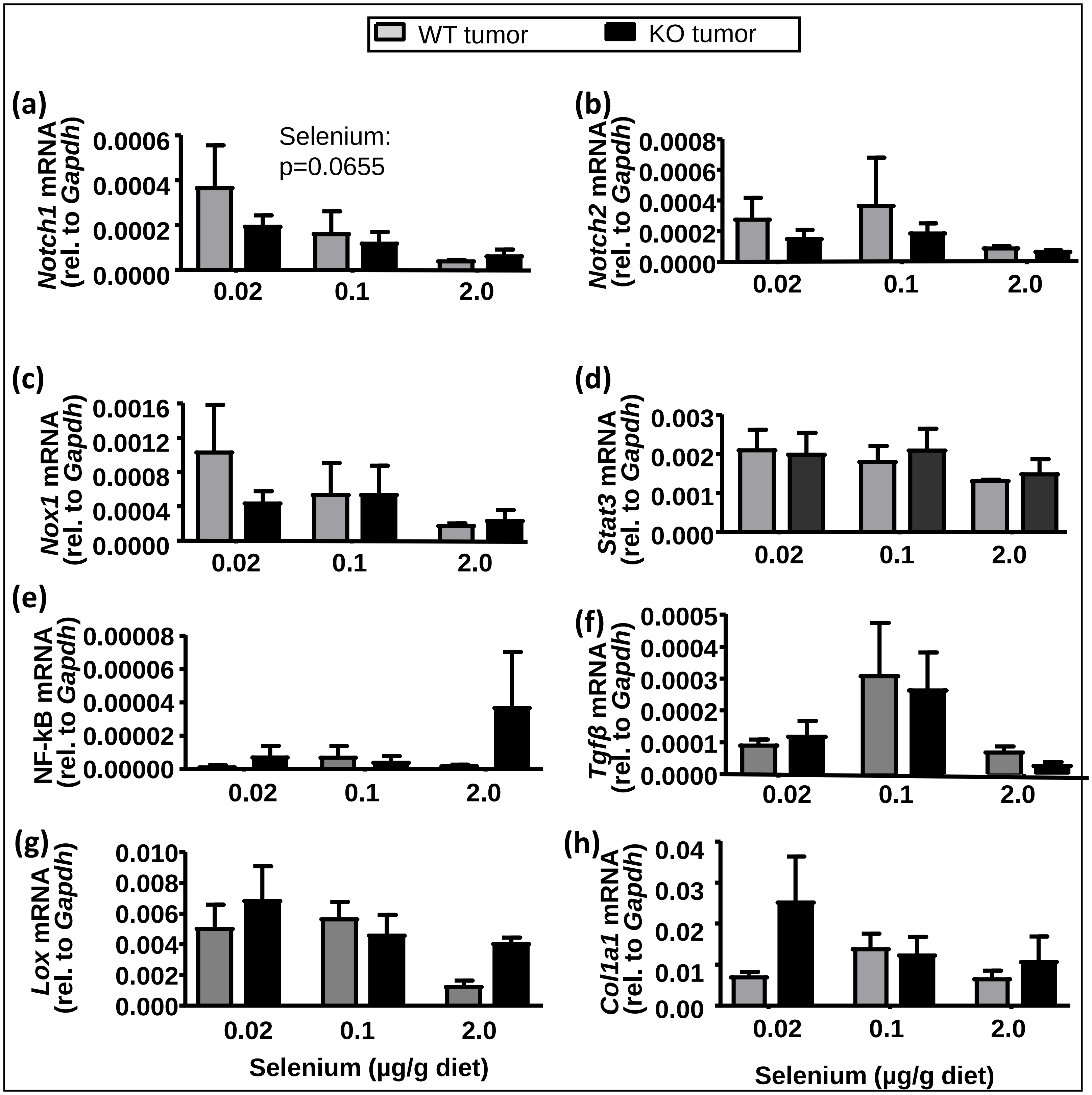

Figure S7. mRNA expression of Wnt/β-catenin-related crosstalk signaling in colon tumors of AOM/DSS-treated animals. Mean (N=4) + SEM, 2-way ANOVA, followed by Tukey's *post-hoc* analyses to compare KO vs WT by diet.
